# Automated vs. manual coding of neuroimaging reports via natural language processing, using the international classification of diseases, tenth revision

**DOI:** 10.1016/j.heliyon.2024.e30106

**Published:** 2024-05-07

**Authors:** Alexander M. McKinney, Jessica A. Moore, Kevin Campbell, Thiago A. Braga, Jeffrey B. Rykken, Bharathi D. Jagadeesan, Zeke J. McKinney

**Affiliations:** a Department of Radiology, University of Miami-Miller School of Medicine, Miami, FL, USA; b University of Minnesota, St. Paul, Minnesota, USA; c HealthPartners, St. Paul, Minnesota, USA; d Department of Radiology, University of Minnesota School of Medicine, Minneapolis, MN, USA; e Departments of Radiology and Neurosurgery, University of Minnesota School of Medicine, Minneapolis, MN, USA; f HealthPartners Occupational and Environmental Medicine Residency, Minneapolis, MN, USA; g University of Minnesota School of Public Health, Minneapolis, MN, USA; h HealthPartners Institute, Minneapolis, MN, USA

**Keywords:** ICD-10, Coding, Neuroradiology, Impact, Relevance

## Abstract

**Objective:**

Natural language processing (NLP) can generate diagnoses codes from imaging reports. Meanwhile, the International Classification of Diseases (ICD-10) codes are the United States' standard for billing/coding, which enable tracking disease burden and outcomes. This cross-sectional study aimed to test feasibility of an NLP algorithm's performance and comparison to radiologists' and physicians' manual coding.

**Methods:**

Three neuroradiologists and one non-radiologist physician reviewers manually coded a randomly-selected pool of 200 craniospinal CT and MRI reports from a pool of >10,000. The NLP algorithm (*Radnosis, VEEV, Inc., Minneapolis, MN*) subdivided each report's Impression into “phrases”, with multiple ICD-10 matches for each phrase. Only viewing the Impression, the physician reviewers selected the single best ICD-10 code for each phrase. Codes selected by the physicians and algorithm were compared for agreement.

**Results:**

The algorithm extracted the reports' Impressions into 645 phrases, each having ranked ICD-10 matches. Regarding the reviewers' selected codes, pairwise agreement was unreliable (*Krippendorff α* = *0.39-0.63*). Using unanimous reviewer agreement as “ground truth”, the algorithm's sensitivity/specificity/F2 for top 5 codes was *0.88/0.80/0.83*, and for the single best code was *0.67/0.82/0.67.* The engine tabulated “pertinent negatives” as negative codes for stated findings (e.g. “no intracranial hemorrhage”). The engine's matching was more specific for shorter than full-length ICD-10 codes (*p* = *0.00582x10*^*−3*^).

**Conclusions:**

Manual coding by physician reviewers has significant variability and is time-consuming, while the NLP algorithm's top 5 diagnosis codes are relatively accurate. This preliminary work demonstrates the feasibility and potential for generating codes with reliability and consistency. Future works may include correlating diagnosis codes with clinical encounter codes to evaluate imaging's impact on, and relevance to care.

## Abbreviations

**ACR**: American College of Radiology
**AI**: Artificial Intelligence
**AIF**: Actionable Incidental Finding
**CPT**: Current Procedural Terminology (CPT)
**EHR**: electronic health record
**ICD**: International Classification of Diseases
**NLP**: Natural Language Processing
**PIF**: Pertinent Imaging Finding
**PNF**: Pertinent Negative Finding
**RFE**: Reason For Exam
**WHO**: World Health Organization

## 1 Introduction

The International Classification of Diseases (ICD) was developed in 1893 to classify mortality causes; since 1948 (6th revision), its scope was expanded by the World Health Organization (WHO) over subsequent revisions to also include morbidity and ultimately to track diseases across populations [[1], [2]]. In 2015, ICD-10 went live in the United States, being mandated by the government for the use of medical coding [[3], [4], [5]]. This update allowed a five-fold increase in possible diagnosis codes, a greater depth of disease characteristics, laterality, and disorder subtypes [[5], [6], [7]]. However, it introduced concerns that more codes could lead to lower coder productivity and specificit y [[7], [8], [9], [10]]. Of note, this is separate from the Current Procedural Terminology (CPT) codes used to bill for a procedure, which denote the anatomic site, modality, use of contrast, etc.; however, CPT codes are not typically useful for morbidity/mortality tracking [[9], [10], [11]].

As ICD codes can be used to follow diagnoses from care episodes, they can, in theory, be used to follow diagnoses from imaging, and derive impact [[9], [10], [11]]. However, while the healthcare industry has weathered the transition from ICD-9 (3–5 characters) to ICD-10 codes (3–7 characters), coding issues remain for imaging [[10], [11]]. Imaging can be particularly susceptible to “avoidable denials”, as most practices use staff or contracted billing services; the conversion to *structured* data from the often free text form of imaging reports has been suggested as a method to improve appropriate billing, particularly via natural language processing (NLP) or other data science methods [[9], [10], [11], [12]]. This has led to calls for standardization, and even homogenization, of ICD codes for structured imaging reports to improve both outcome tracking and billing, based on providing relevance to a clinical care episode [[10], [11], [12], [13]]. One promising aspect for imaging report coding standardization is that only a small minority of codes pertain to the most common imaging exams; it is estimated that <3 % of ICD-9 and <10 % of all possible ICD-10 codes are applicable to >90 % of imaging claims [[9], [10]]. In theory, focusing on this minority enables optimization of ICD-10 codes to track eventual outcomes from pertinent imaging findings (PIF's), while confirming relevance of the reason for exam (RFE) orders [[9], [10]]; however, there remains a gap in research that proves outcomes from imaging due to its inherent tethering to a single time point of care [[14], [15]]. Early works have thus tried to focus on the most actionable portion, the *Impression* section, of imaging reports to derive immediate impact, for the purposes of disease surveillance, quality, and to improve the specificity and recall of NLP algorithms [[16], [17], [18], [19], [20]]. Hence, in an effort to improve the reliability and quality of reported codes so impact can be assessed in the future, this study sought to evaluate whether an NLP algorithm could automate the process of assigning codes to the Impressions of neuroimaging reports, and to assess the interobserver reliability of physician coding via neuroradiologists.

An important background point is that the vast majority of radiologists do not code their own reports, a practice quite familiar to, and performed by, >90 % of primary care physicians [[21], [22], [23]]. Early, limited works have advocated for standardized coding practices to provide consistency in documentation to payors to facilitate legitimate reimbursement; however, considerable coding disagreement between physicians exists, a phenomenon scantly evaluated in imaging research [[23], [24], [25], [26]]. Market solutions do exist to code imaging reports in an automated fashion; however, these either: are not devised for imaging (i.e. retrofit to radiology), use ICD-9, extract only diagnoses (not ICD codes), used small datasets (<1000 reports), or did not use coding-trained neuroradiologists as “ground truth” [[27], [28], [29], [30]]. Hence, this novel work is one of the first to assess the coding homogeneity of manually selected ICD-10 codes between physicians reviewing the imaging reports, thereafter comparing the NLP's auto-coding to the reviewer consensus.

## 2 Methods

This cross-sectional study was deemed exempt from review by the institutional review board of two institutions, since the database inquiries obtained were: a) for the purpose of quality initially, and b) as the database was comprised of data in which the PHI had already been removed (and age of patients >89 years'), thus containing only alias data; subsequently, patients' names and identifiers were swapped with aliases. Using a tertiary medical center's radiology information system, 200 total neuroimaging reports of the head, neck, and spine were randomly selected from a pool of 10,488 adult CTs and MRIs performed over a 6 month period of 01/01–06/30/2016, which contained 645 Impression phrases. All were semi-structured with headings common to imaging reports, such as “Findings” or “Impression,” though the phraseology of sections varied, for example “Conclusions” instead of “Impression.” All text within structured headings was written in prosaic text (English language consistent with imaging dictations), other than to the extent that the Impressions may have contained measurements, numbered points, etc. The randomly-selected reports were then used for both the manual code selection by the physician reviewers, and for the automated NLP-based coding.

### 2.1 Manual ICD-10 coding

The physician reviewers (3 board-certified neuroradiologists, 1 occupational and environmental medicine) were assigned the same 200 neuroimaging reports (finalized by a sample of 29 radiologists from three different groups) and asked to independently assign an ICD-10 code to each of the Impressions’ phrases. Each reviewer received 1 h of training on ICD-10 code selection, with another hour of instruction on using a web-based interface to search an ICD-10 database by diagnosis, code, or descriptions of codes; they assigned a single code to phrases parsed by the NLP engine (Fig. 1a). This interface for manually assigning codes to NLP engine phrases was designed for the purposes of this validation, with capabilities for searching all ICD-10 codes akin to EHR interfaces that patient-facing providers utilize to associate diagnostic codes to their encounters. The three radiologist reviewers did not have dedicated experience in ICD-10 coding beyond the 1 h spent; however, the non-radiologist reviewer did have prior experience in code selection as used for their daily clinical practice. Each reviewer independently selected a single “best” ICD-10 code thought most relevant to each of the Impression phrases, with only the text, modality, and body part visible for context during review. For “negative”/“normal” reports, a reviewer could pick either a specified code as a “pertinent negative finding” (PNF) based on the Impression (e.g. if the report was negative, picking a code for “hydrocephalus, unspecified” as a PNF) or, if they did not feel an ICD-10 could be assigned (e.g. “unremarkable” or “normal”), they were to state there was “no applicable ICD-10 code” for that Impression phrase.

**Fig. 1a:**
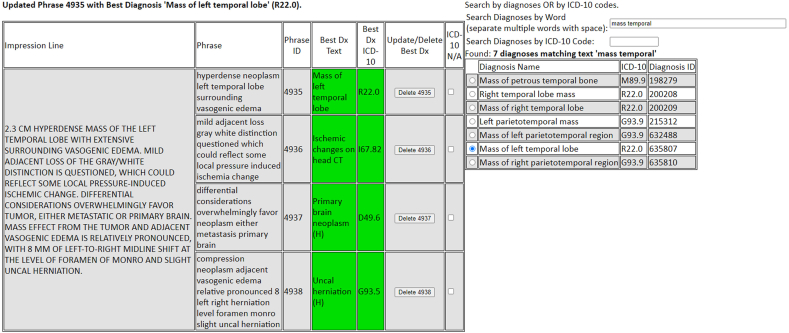
Example of the user interface used for “manual coding”; in this patient, a noncontrast CT report's Impression section (note: patients' identifiers swapped with alias data) describes a left temporal lobe mass. The user's entered search for “temporal mass” yielded several possible codes (*right*), and then the user selected “R22.0” (‘mass of left temporal lobe’) for this finding (*middle, in green*). Three other such searches for phrases by the user yielded codes for “ischemic changes on head CT” (I67.82), “primary brain neoplasm” (D49.6), and “uncal herniation” (G93.5), which are highlighted in *green* as well; the user subsequently attached these 4 manually-selected codes to the report. (For interpretation of the references to colour in this figure legend, the reader is referred to the Web version of this article.)

### 2.2 Automated report segmentation and ICD-10 coding

The training neuroimaging report dataset of 10,488 reports was scrubbed for protected health information (PHI, including “aliases” being substituted for any identifiers regarding patient name, exam codes, site etc.), being subsequently used to evaluate the NLP algorithm's performance. It was obtained from a hybrid tertiary care academic and community system that consists of five hospitals with several outlying urban, suburban, and rural ambulatory care centers. The training dataset contained reports from three distinct practicing radiology groups that comprised a total of 46 radiologists, with 9 (private practice), 14 (academic tertiary care), and 23 (hybrid practice) radiologists from each group, respectively. Utilizing a contextualized bag-of-words approach, as utilized in other preliminary works to evaluate medical records (albeit not for diagnostic codes), the NLP engine (*Radnosis, VEEV, Inc., Minneapolis, MN*) processed the Impression sections of the radiology reports into Impression “lines” that corresponded to the bulleted or numbered elements of a radiology report's Impression. These Impression lines were then further parsed into separate Impression “phrases” of distinct imaging findings based on context identifiers [20,[31], [32]]. The phrases were simplified via removal of most non-alphanumeric characters, with word replacement based on grammar rules and clinical synonyms. The engine classifies each phrase as positive (presence of a finding) or negative (absence of a finding) for a particular diagnosis code. “Normal” reports were treated as a PNF, as dictated by the Impression (e.g. “no intracranial hemorrhage”), being classified as a negative for that particular ICD-10 code. Of note, current available vendor technologies do not capture PNF's [[27], [28], [29], [30]].

As the algorithm generates a list of matched ICD-10 codes for each Impression phrase, it can attach multiple codes to an Impression via an odds ratio-like approach to weight the possible diagnoses for each phrase, with a “*match score*” for each potential matching code; the highest corresponds to the best code match (Fig. 1b). This score takes into account body part and exam type (such as per the CPT code) when a phrase is weighted to optimize the specificity for matching a diagnosis code; examples of how this augments the engine's matching capability are if anatomical names utilize the same or similar names for different body parts (e.g. “glenoid”, “ala”, “malleal” vs. “malleolar”, etc.), or when pathology is not specific to a body part (e.g. “aneurysm”, “mass”, “nodule” etc.). The “match score” is also based on various language-related factors, including the number of words matched between the phrase and a diagnosis, phraseology alignment between a phrase and diagnosis, frequency of certain words in the diagnosis database, and the weighting of words based on clinical context (such as whether a word indicates an anatomic versus a pathologic finding). Although the match scores theoretically range from zero to infinity (akin to an odds ratio), a match score's magnitude corresponds with the phrase, since a potential match of greater length would be scored higher.

**Fig. 1b:**
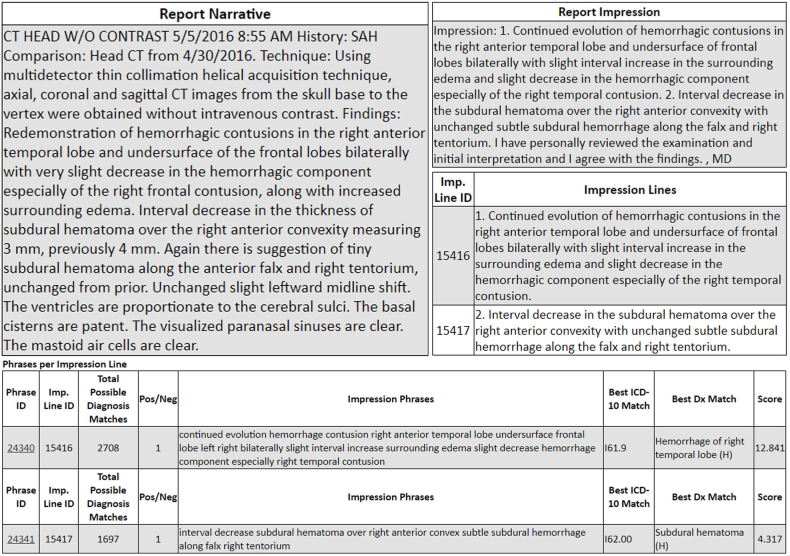
Sample NLP-generated diagnosis codes from a noncontrast CT report. The algorithm separates the Impression of the report (*top right*) into Impression phrases of the report (*middle, right*), which are separated from the rest of the report narrative (*top left*). The generated ICD-10 codes and the ranking/weighting of the likelihood of a “match” are simultaneously provided (*bottom*), with the single best ICD-10 match for that phrase; clicking on the “phrase ID” (*leftmost column, bottom*) provides a list of the other, lower ranked possible matches for that phrase.

### 2.3 Statistical analysis

As it was not possible to establish a definitive “gold standard”, a “ground truth” was necessary so other definitions for algorithm performance could be derived. For the purposes of this cross-sectional study, the “ground truth” was considered to be when all four reviewers agreed unanimously on a single code for a given phrase, as this approach is most conservative in representing expert consensus on which code is the “best” for a given phrase. By this logic, a “true positive” (TP) for algorithm performance was when all four reviewers unanimous agreed on the code corresponding to a phrase, and the NLP engine found the same code; ergo, a “false negative” (FN) was if the engine did not find the same code as that chosen unanimously. However, delineating a “false positive” (FP) or “true negative” (TN) required a more nuanced approach to evaluate performance, since if, for example, only two reviewers agreed, there is no certain “ground truth”, and the NLP engine's performance may be construed as either a TN or FP. This complex situation involved creating a *match score* threshold (prior paragraph), which was defined as the average match score of the “ground truth” (TP matches) results for each “top x” rank (i.e. top 1, 5, 20, 100). When all “top x” match scores were *below* the defined threshold (e.g. for the top 20 matches, the top 20 NLP match scores were below that threshold), the engine's results for these phrases were deemed TN. In the consideration for a FP of engine's performance, when at least one of the “top x” results was above the threshold, the result for that phrase was considered FP (as unanimous agreement did not exist); hence, the threshold for a match score to be considered TN or FP was based on the average of the match scores generated by the engine in TP cases (i.e., those with unanimous reviewer agreement). These definitions are provided in Table 1.

**Table 1:** Definition of true positive/negative (TP/TN), false positive/negative (FP/FN).

Engine Result	Reviewer Agreement: If All 4 Reviewers Agree	Reviewer Agreement: Some/None Agree
**Positive** Result scored within ranked results (1, 5, 20, 100) at/above the threshold match score.	**True Positive (TP)** The agreed upon code for all reviewers was in the top x results.	*False Positive (FP)* At least one code in the top *x* results was above the threshold match score.
**Negative** Result scored within the ranked results (1, 5, 20, 100) below the threshold match score.	*False Negative (FN)* The code agreed upon by all reviewers was not in top *x* results.	*True Negative (TN)* All codes in the top *x* results were below the threshold match score.

Threshold score = average score of TP results within the included ranked results (1, 5, 20, 100).

Reviewer interobserver agreement and engine performance relative to reviewers was evaluated for the codes' lengths, at lengths of 3 (3L), 4 (4L), and 7 (7L) characters; the “dot” within an ICD-10 code was excluded. Additionally, the engine's multiple best matches of codes were compared with those having *unanimous reviewer agreement*, with inclusions of the top 1, 5, 20, and 100 codes provided by the engine. Agreement regarding the ICD-10 codes assigned by the four reviewers was calculated; Cohen's kappa (κ) and Fleiss' κ (for >2 reviewers) were used to evaluate interrater reliability of the reviewer's capacity to determine the presence of any applicable ICD-10 code in a *binary* fashion (as to whether or not there was an applicable code) for each of the 645 Impression phrases. Krippendorff's alpha (α) assessed interrater reliability for the 645 phrases, (significance set to *p ≤ 0.05*, [33]). The engine's performance was then evaluated using sensitivity, specificity, and F2 as compared to the “ground truth” (“gold standard”) of unanimous agreement; note that for data models, F2 is often a preferred alternative to the traditional measurement of accuracy, where F2 is the weighted harmonic mean of the precision and recall of a model, albeit prioritizing recall over precision. Statistical comparison of the engine's performance parameters using varying lengths of ICD-10 codes for comparison were conducted. All statistical analyses were performed using Microsoft Excel (*Microsoft Corporation, Redmond, WI*) and R statistical software [34].

## 3 Results

The 200 reports resulted in a total of 123 CTs and 77 MRIs (Table 2), varying in terms of patient setting (Table 3). All three radiology groups from the larger training dataset were represented in the 200 report sample, with 29 total radiologists (63.0 % of 46 total radiologists), comprised of 7/9 (77.8 %), 8/14 (64.3 %), and 14/23 (60.9 %) from each radiology group, respectively. Other than being adults, the distribution of ages and sex for the 200 reports were not known, as the identifiers were removed. In total, the Impression sections of the reports totaled 380 Impression lines, having a total of 645 resulting Impression phrases (*mean 3.28 phrases/report*), as shown in Table 3.

**Table 2:** The numbers of and characteristics of the 200 total reviewed radiology reports are provided in terms of modality, body part, and presence/absence of intravenous contrast.

Type of Radiology Report	Number of Reports	Number of Impression Lines	Number of Impression Phrases
CT Cervical Spine w/o Contrast	11	18	30
CT Head w/o & w Contrast	3	7	9
CT Head w/o Contrast	73	131	191
CT Lumbar Spine w/o Contrast	4	11	28
CT Maxillofacial w/o Contrast	6	11	15
CT Sinus w/o Contrast	5	7	7
CT Soft Tissue Neck w Contrast	11	22	33
CT Soft Tissue Neck w/o Contrast	1	2	5
CT Thoracic Spine w Contrast	1	3	8
CT Thoracic Spine w/o Contrast	4	10	16
CTA Angiogram Head	1	2	7
CTA Angiogram Head Neck	3	9	9
MR Brain Stroke w Contrast	1	4	11
MR Stroke w/o & w Contrast	3	11	23
MR Brain w/o & w Contrast	31	53	95
MR Brain w/o Contrast	6	8	12
MR Cervical Spine w/o Contrast	5	7	20
MR Lumbar Spine w Contrast	1	1	1
MR Lumbar Spine w/o & w Contrast	5	17	27
MR Lumbar Spine w/o Contrast	11	24	50
MR Sacrum and Coccyx w/o Contrast	1	5	15
MR Neck w/o & W Contrast	1	1	6
MR Thoracic Spine w/o & w Contrast	1	2	3
MRA Angio Head w/o Contrast	6	7	11
MRA Angio Neck w/o & w Contrast	5	7	13
**Total**	**200**	**380**	**645**

**Table 3:** The patient setting is provided, with numbers of patients’ reports in each setting, and the number of Impression phrases total for each setting.

Patient Setting	Number of Reports	Number Impression Phrases
Emergency	63	167
Inpatient	73	293
Outpatient	64	185
**Total**	**200**	**645**

**Fig. 2:**
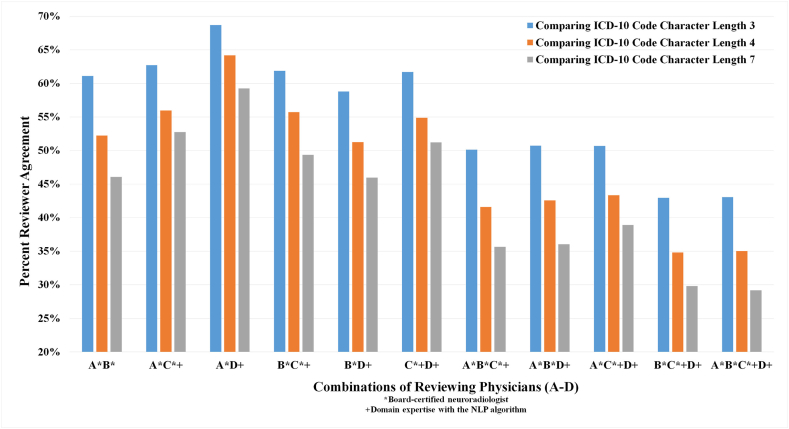
Figure shows the percent agreement amongst reviewers that assigned an ICD-10 code. The degree of agreement changes with the ICD-10 code character length. *Board-certified neuroradiologist; +Domain expertise with the NLP algorithm.

Each reviewer spent between 5 and 8 h assigning ICD-10 codes to all 645 phrases, (average 6.375 h), averaging 35.6 s per phrase. They unanimously found that 31/645 (4.8 %) of the Impression phrases did not have an applicable code, usually occurring in the situation of a terse Impression consisting of words such as "normal" or "unremarkable" in isolation, without an accompanying description of PIF's (positives) or PNF's (negatives). Between reviewers, there was moderate-high pairwise agreement for a range of Cohen's kappa (κ *range* = *0.42-0.68, each p < 0.05*), and moderate Fleiss' kappa of 0.546 (each *p < 0.05***)** for all 4 reviewers (Table 4). Regarding reports in which all four reviewers found applicable codes and assigned them (497/645 phrases, 77.1 %), the percent agreement on an exact code picked decreased as code length increased (Fig. 2), showing increased matching agreement with shorter lengths. Conversely, in 148/645 (22.9 %) there was *not unanimity* as to whether there was an applicable code (i.e. at least one reviewer could not find an applicable code in 22.9 %*)*.

**Table 4:** The interrater/reviewer reliability/agreement on applicability (Kappa) of Coding selection (alpha) of ICD-10 codes of varying character lengths (3L, 4L, 7L) is provided. Note that the interrater agreement generally decreased with increasing code length.

Reviewers (A-D)	Kappa^^^ (κ) [agreement on a code's applicability]	Krippendorff's Alpha (α) [agreement on a specific code or no code]		
		Length 3L	Length 4L	Length 7L
A* and B*	0.42	0.51	0.45	0.39
A* and C*^+^	0.63	0.57	0.51	0.48
A* and D^**+-**^	0.65	0.63	0.59	0.55
B* and C*^+^	0.47	0.54	0.49	0.43
B* and D^**+-**^	0.45	0.51	0.45	0.40
C*^+^**and D**^**+-**^	0.68	0.57	0.52	0.49
A*, B*, C*^+^	0.51	0.54	0.48	0.44
A*, B*, **D**^**+-**^	0.51	0.55	0.49	0.45
A*, C*^+^, **D**^**+-**^	0.56	0.59	0.54	0.51
B*,C*^+^, **D**^**+-**^	0.52	0.54	0.48	0.44
A*, B*, C*^+^, **D**^**+-**^	0.55	0.55	0.50	0.46

*Board-certified neuroradiologist; +Board-certified clinical informaticist; -Board-certified Occupational and Environmental Medicine physician with coding experience; ^Either Cohen's kappa (used for only two reviewers), or Fleiss' kappa (used if there were 3–4 reviewers).

The reviewers' manually-selected codes had greater agreement at length 3L (0.51 ≤ α ≤ 0.63) as compared to lengths 4L (0.45 ≤ α ≤ 0.59) and 7L (0.39 ≤ α ≤ 0.55), as shown in Table 4; the mean Krippendorff's alpha for all combinations of reviewers being statistically significant (each *p ≤ 0.05*). The reviewers unanimously agreed on specific manually-selected codes compared at length 3L for 214 phrases, at 4L for 174 phrases, and at 7L for 145 phrases (a list of the codes is provided in Table 5). There was moderate variability and heterogeneity in ICD-10 code assignment, with considerable disagreement amongst reviewers as to whether a code was applicable to assign to some of the 645 Impression phrases. Notably, higher degrees of agreement were a) between two neuroradiologists (one with informatics certification), and an occupational and environmental medicine informaticist.

**Table 5:** A complete list of the ICD-10 codes upon which there was unanimous reviewer agreement, with codes compared at varying character lengths.

Length 3 (n = 60; 214 phrases)	Length 4 (n = 74; 174 phrases)	Length 7 (n = 74; 145 phrases)
*C71*	*C77.0*	*C77.0*
*C77*	*C79.3*	*C79.31*
*C79*	*C85.8*	*C85.89*
*C85*	*D17.0*	*D17.0*
*D17*	*D17.7*	*D17.79*
*D18*	*D18.0*	*D18.09*
*D32*	*D32.9*	*D32.9*
*D49*	*D49.6*	*D49.6*
*E04*	*E04.1*	*E04.1*
*G06*	*G06.1*	*G06.1*
*G08*	*G08*	*G08*
*G31*	*G31.9*	*G31.9*
*G37*	*G37.9*	*G37.9*
*G91*	*G93.1*	*G93.1*
*G93*	*G93.4*	*G93.41*
*G95*	*G93.5*	*G93.5*
*H70*	*G93.6*	*G93.6*
*I60*	*G93.8*	*G93.89*
*I61*	*G95.1*	*G95.19*
*I62*	*H70.9*	*I60.9*
*I63*	*I60.9*	*I61.5*
*I65*	*I61.5*	*I61.9*
*I66*	*I61.9*	*I62.00*
*I67*	*I62.0*	*I62.9*
*I72*	*I62.9*	*I63.512*
*I77*	*I63.5*	*I63.8*
*J03*	*I63.8*	*I63.9*
*J32*	*I63.9*	*I65.02*
*J34*	*I65.0*	*I65.21*
*J36*	*I65.2*	*I65.22*
*J39*	*I66.1*	*I65.29*
*K11*	*I67.1*	*I67.1*
*K38*	*I67.8*	*I67.83*
*M25*	*I72.6*	*I72.6*
*M46*	*I77.1*	*I77.1*
*M47*	*I77.7*	*J03.90*
*M48*	*J03.9*	*J32.0*
*M50*	*J32.0*	*J34.2*
*M51*	*J34.2*	*J36*
*M53*	*J36*	*J39.0*
*M70*	*J39.0*	*K11.5*
*M99*	*K11.5*	*K38.9*
*N20*	*K38.9*	*M25.459*
*Q04*	*M25.4*	*M46.20*
*Q28*	*M46.2*	*M47.812*
*Q76*	*M47.8*	*M48.02*
*R22*	*M48.0*	*M48.04*
*R59*	*M50.2*	*M48.06*
*R60*	*M51.2*	*M50.20*
*R93*	*M53.2*	*M51.26*
*S00*	*M70.6*	*M53.2X1*
*S01*	*M70.7*	*M99.81*
*S02*	*M99.8*	*M99.82*
*S06*	*N20.0*	*M99.83*
*S12*	*Q04.6*	*N20.0*
*S22*	*Q28.3*	*Q04.6*
*S32*	*Q76.4*	*Q28.3*
*S72*	*R22.0*	*Q76.49*
*Z96*	*R60.0*	*R22.0*
*Z98*	*R93.0*	*R60.0*
	*R93.7*	*R93.0*
	*R93.8*	*R93.7*
	*S00.0*	*R93.8*
	*S01.8*	*S00.03XA*
	*S02.1*	*S01.81XA*
	*S02.8*	*S02.119A*
	*S02.9*	*S02.92XA*
	*S06.2*	*S06.2 × 9A*
	*S12.9*	*S12.9XXA*
	*S22.0*	*S22.009A*
	*S32.0*	*S22.080A*
	*Z96.8*	*Z96.89*
	*Z98.2*	*Z98.2*
	*Z98.8*	*Z98.890*

**Fig. 3:**
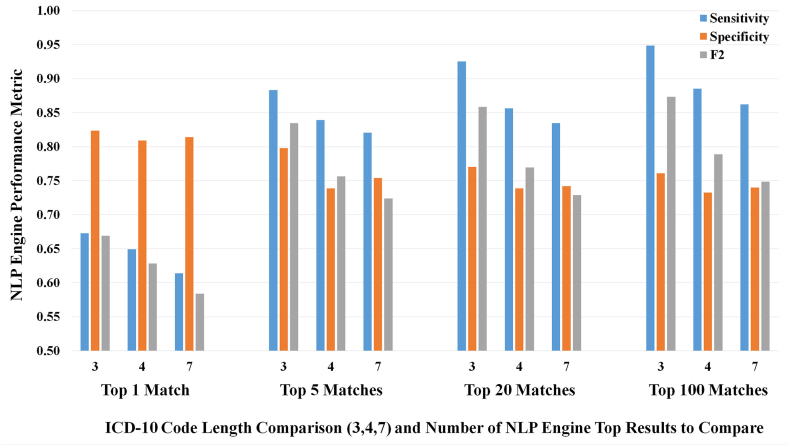
Figure depicts the Sensitivity, Specificity, and F2 values of the NLP algorithm when comparing different numbers of codes (from the single top code to the top 100 generated ICD-10 Codes at varying character lengths (i.e. at ICD-10 code lengths of 3, 4, and 7 characters). Generally, shorter lengths had higher degrees of agreement.

The NLP engine was fast, requiring an average of 0.06 s per phrase to generate all potential matched codes. The algorithm's sensitivity in correctly assigning the same ICD-10 codes as the *unanimous* reviewer choices was inversely proportional to the length of the code (Fig. 3); this sensitivity increased as the number of included matches increased (top 1 code to top 100). Regarding the “match scores” for each code chosen by the NLP, the range of scores for the 29,594 extracted phrases in the 10,488 report database ranged from 0.20 (extremely low likelihood of a match) to 81.36 (extremely high likelihood). For the entire training dataset, the NLP engine generated an average of 937.0 codes per impression phrase (range 32-5039), where the automated generation of codes for 29,594 phrases required a total of 29.2 min (an average of 0.06 s per phrase). The average match score of TP results for the top 5 ranked results at varied lengths of ICD-10 codes (described further in the following paragraph), were 7.98, 7.42, and 7.68 for code lengths of 3L, 4L, and 7L respectively, wherein the match scores ranged from a minimum of 0.018 to a maximum of 53.06.

Regarding the specificity of the NLP engine for code selection, the highest specificity (82 %) was observed at length 3L and via only assessing the engine's top code; the lowest specificity (73 %) was observed at length 4L and including the top 100 codes from the engine. In general, the specificity of the algorithm decreased gradually as the number of included codes increased (top 1 to top 100). Overall, gains in sensitivity and F2 were observed *by increasing the number of included NLP-generated codes from the top 1 to top 5*, with fewer gains when including additional codes after the top 5 codes. The optimal performance was at code length 3L; the top 5 codes had a sensitivity of 88.3 %, a specificity of 80.0 % and an F2 of 82.7 %, per reviewer consensus; the 7L length had lower performance (82.1 %, 75.4 %, 72.9 %, respectively), with significantly different sensitivity and specificity (*p* = 5.82*10^*−6*^) between the 3L and 7L lengths.

## 4 Discussion

The United States' mandated use of ICD-10 coding affects almost all healthcare systems; ICD-10 is a cornerstone of medical billing reimbursement that is increasingly utilized to also assess outcomes and quality. However, the numerous rules associated with its coding have led to a complex billing environment, with challenges in measuring outcomes, resulting in uncertainties even amongst experts [[3], [4], [5], [6], [7], [8], [9], [10], [11], [12], [13]]**.** This has led to works that show differences in ICD-10 coding proficiency by level of experience, training, and support with subsequent consequences; hence a need for homogenization [[21], [22], [23], [24], [25], [26]]. As regards to the field of radiology, this phenomenon has led to a growing potential space in the billing/coding realm as well as in the arena of value/quality; while imaging has a high contribution to financial margin and a direct effect on care via objective diagnoses, >90 % of radiologists do not attest to their report's codes. In contrast, the vast majority of primary care doctors do attest to them. Radiologists have yet to adopt such coding stewardship, limiting the prospects for optimizing legitimate billing and outcomes works. Ergo, given the coding variability between physicians, this study set out to test if an NLP engine could match the radiologists' consensus of codes, which it did fairly well, while the physicians at best only mild-moderately correlated with each other's choices.

Hence the need to validate, and therafter enable, automated coding software for radiologists' attestation, with a level of consensus, so the field can move forward in both outcomes research and appropriate billing. On the subject of outcomes (a primary focus of the WHO), despite various ICD-10 initiatives leading to improved disease surveillance and coding/billing workflows, longitudinal outcomes related to ICD-10 have been slow to progress, related to deficits in physician training and converting prior ICD-9 data ([6], [7], [8], [9], [10], [14], [15], [35], [36], [37], [38], [39], [40], [41]). Even more pointedly in the imaging field, there is a strong, traditional reliance on free-text narratives with unstructured *Findings* and *Impression*s sections of their reports; this phenomenon has often been cited as a barrier to eliciting direct impact (a more immediate measure than outcome) of imaging procedures. Such prior studies have shown accordingly that diagnoses conveyed in the Impression are usually real and actionable, whereas NLP algorithms based on the entire report (*Indication, Technique, Findings, Impression*) may be less specific in detecting actionable findings; notably, a combination of nonspecific language, multiple similar findings, and the testing dataset's treatment of “normals” (or their prevalence in the training dataset) can affect an engine's performance [[16], [17], [18], [19], [20], 24, [27], [28], [29], [30], [42], [43]]. Accordingly, NLP-based algorithms for coding or deriving diagnoses from reports have shown greater accuracy, specificity, and recall when focusing on primarily the *Impression* section rather than an entire free text report [[16], [17], [18], [19], [42], [43]]. Thus, this study focused primarily on the Impression section's data to ascertain the “ground truth” of a radiologist's opinion, which is usually where the PIF's reside that are most likely to be “actionable”; actionable incidental findings (AIF's, e.g. a lung nodule to be followed) are also usually summarized in this section. Once identified, the *Impact* of a single PIF, an entire report, or even the cost-benefit impact of a modality on a larger scale for a certain disorder becomes measurable, perhaps serving as a waypoint to approximate outcomes [[8], [9], [10], [11], [35], [36], [37], [38], [39]]. One such use case has been early examples of ICD-10-based cost-benefit analyses on advanced imaging (CT or MRI) for spinal degenerative disease and stenosis, which found that imaging has the highest cost of nonoperative procedures or therapy in symptomatic cases, followed by epidural injections and opioid prescriptions, of import given the recent opioid pandemic [[39], [40]].

As the WHO's intent is to track morbidities and outcomes, the promise of ICD-10 at longer code lengths (up to 7L) is greater granularity, due to a larger number of codes (where ICD-9 was inadequate), but this can create more variability in coding, with lower productivity or specificity [**5**–**11,35**–**41**]. In this regard, our finding of mild-moderate interrater reliability via manual coding with lower reliability at longer code lengths further highlights known issues with reliability and homogeneity in code assignment between coding-trained physicians. In this imaging-based study, the algorithm's top 5 codes had higher sensitivity (88 %) and specificity (80 %) at the shorter code length 3L, suggesting that shorter code lengths can be relatively accurate for radiology coding. One reason for this may be because the first 3 characters indicate a general category, while greater lengths indicate specific anatomic sites, severity, sequalae, chronicity, etc., thus increasing variability as the code length increases. Also, radiologists are often not privy to detailed descriptions of a disease outside of the ordered RFE, so manual coding may cause them to variably select the longer code lengths based on presumptions, thus causing variability. This may be another reason to automate and standardize imaging coding; when dedicated clinical data is lacking, selecting the general code category may minimize individual variation. Importantly, manual coding variability could worsen with the advent of ICD-11 (adopted by the WHO in 2019); ICD-11 has more codes and is touted to be better suited for incorporating digital health innovations and assessing outcomes due to its greater granularity and ability to integrate with other coding systems [44]. Ergo, ICD-10 homogenization should be prioritized and validated for radiology.

Hence a need to move towards auto-coding via validated algorithms, particularly in imaging to preserve the productivity of the coder or even a dictating radiologist; health systems in the United States are financially dependent on imaging, often in the range of 35–40 % of their revenue (**45**). However, given radiologists' historical lack of adoption to code reports, the bar is high for the educational and acceptance fronts. Meanwhile, given various policy changes over time, the legacy approach of connecting certain ICD-10 to CPT codes for advanced imaging orders (MRI, CT, or PET) has led to increasingly unsupported claims (referred to as “write-off's”) if an order's reason for exam (RFE) is not corroborated by recent clinical encounter-based codes or symptoms [[10], [11], [25], [26], 41, 45]. Common examples of unrevealing RFE's in neuroradiology are “*Headache*” (ICD-10 code R51), “*Altered mental status, unspecified*” (R41.82), and “*Dizziness, giddiness*” (R42); such nonspecific codes are increasingly scrutinized by payors due to a lack of medical necessity, relevance to a care path, or specificity [[10], [11], [25], [26], 45]. Similarly, a RFE consisting solely of “R/O …” (rule out) is unlikely to be reimbursed. Thus, additional, alternative, or standardized ICD-10 codes could lead to better coding accuracy, and enable correlation with codes from other clinical visits to delineate both the *relevance* (to an underlying disorder) as well as imaging's direct *impact* on resultant diagnoses, therapy, or morbidity (**41**). In fact, the United States' Center for Medicare & Medicaid Services (CMS) allows the addition of relevant data or codes from a patient record (i.e. not part of the ordered RFE), for the purposes of proper procedural reimbursement of imaging [12, [46], [47]].

Hence, this study supports the use of an NLP engine to aid in homogenizing ICD-10 coding via automating the suggestion of pertinent codes, as such codes may provide the impact or relevance of imaging if optimized by structured reporting and standardized nomenclature. Ultimately such codes could, *as envisioned by the WHO*, enable future delineation of outcomes from imaging, retrospectively confirm the *appropriateness* of the performed imaging per the provided RFE, and if carried forward, additionally aid the appropriateness of the next ordered study's RFE (such as when following PIF's). With this aim, preliminary works using ICD codes to reflect impact or outcomes found that actionable data is best communicated concisely within the Impression of structured reports when radiologists follow standardized reporting guidelines (such as thyroid and pulmonary nodules); this optimizes communicating and following PIF's [[16], [17], [18], [19], [20], 48]. Early works using ICD-9 or ICD-10 codes derived from imaging to follow direct impact and morbidity included studying disorders such as intracranial hemorrhage, myocardial infarction, stroke, trauma, gastrointestinal bleeding, seizures, pulmonary or venous emboli, and pneumonia [14, [49], [50], [51], [52], [53]]. Thus, the implementation of NLP and techniques such as deep learning, machine learning, or generative artificial intelligence models can help standardize diagnostic codes for PIF's, in order to prove that imaging serves as an “inflection point” in care pathways, such as in the realm of actionable incidental findings (AIF's); in the future, these techniques could also help delineate or even predict morbidity, mortality, outcomes, or other downstream affects from imaging [[54], [55], [56]]**.**

The question may arise as to why radiologists would be asked to code reports. The concept of “self-coding” arises from the practice being accepted amongst primary care physicians, where the majority select diagnosis codes to bill their visit, and attest to the patient's condition, which typically confirms the relevance of a care episode ([21], [22], [23], [24], [25], [26]). In concept, a radiologist self-coding a report similarly could attest as well, confirming the relevance of the report's diagnoses to other care visits (and imaging appropriateness), including the impact on subsequent care ([25], [26]). A published limitation in primary care self-coding is that >70 % of primary physicians are uncomfortable with their choices of codes, there can be significant disagreement, and most opine that they have suboptimal coding training ([21], [22], [23], [24], [25], [26]). Similarly, based on this study's preliminary results, radiologists' coding their own reports manually may not be optimal (regarding both coding accuracy and productivity), unless validated algorithms aid in “autocoding” reports, in which an algorithm proposes pertinent codes to the radiologist, and the nominally coding-trained physician subsequently attests to the proposed codes.

The primary limitations of this study are its retrospective design and perhaps the physician reviewers' lack of dedicated coding experience, despite training. This lack of extensive coding proficiency is typical of, and mimics most radiology practices, as the vast majority of providers lack in-depth coding experience. Another limitation is the non-homogeneity of the reviewer subspecialty training, albeit not likely significant, as the highest degree of discrepancy was between two board-certified neuroradiologists. This may point to another limitation, the lack of a “gold standard” for the physicians' coding; while a consensus of all four reviewers was considered optimal and a ground truth of sorts, it was difficult to confirm clinically, given the dataset's de-identified nature. Also, regarding the limitations of the dataset, the neuroimaging focus limits generalizability to other body parts; we note that neuroimaging was the focus of this pilot since it is the largest portion of cross-sectional imaging at most imaging sites, and its reimbursement has been diminishing via CPT code bundling, bundled payments (per care episode), multiple procedure payment reduction (MPPR), medical necessity write-off's, denied or untimely coding appeals, and other challenges. As such, clear and concise structured reports in accord with ICD-10 (including acuity, laterality, anatomic site, etc.) can abilitate proper reimbursement ([57], [58]). There was also a limitation in the relatively small number of reports used to compare between physicians, due to the time limitations for physicians; future works using larger datasets, more radiologists, and training in other subspecialties would be needed to confirm if these results translate to other areas. Finally, the NLP engine used herein is preliminary; optimally, it would compare imaging diagnoses' codes to those from clinical encounters' in order to delineate the *impact* of imaging on diagnosing (PIF's) or excluding (PNF's) particular disorders; some early works have shown that marrying NLP to ICD-9 or ICD-10 codes can corroborate encounter diagnoses (20, 41, [50], [51], [52], [53]).

Future directions include using automated, standardized report coding outside of the domain of neuroradiology to ultimately help demonstrate imaging's value, confirm appropriateness, support legitimate reimbursement, and address the issues related to tracking PIF's and AIF's and their related outcomes ([53], [54], [55], 59). Ultimately, using NLP for automated coding could evolve into real-time attestation by radiologists to the proposed codes. This could result in improved coding timeliness and reimbursement if they attest to the appropriate codes while signing their dictated report. Further, validating such automated coding by content experts can serve as a guiderail of sorts to positively harness generative AI models, via limiting them to codes *relevant* to patients' underlying conditions; this is of import given the predisposition of such algorithms to “confabulate” or “hallucinate” data, results, and findings on imaging [[60], [61], [62]].

## 5 Conclusion

This study highlights the variability and lack of concordance between trained physician reviewers when manually assigning ICD-10 codes, whereas the algorithm often agreed with the reviewer consensus, being more accurate at shorter code lengths. This enables future directions that include the ability to demonstrate radiologists' direct impact on (or relevance to) care, as well as potentially using NLP for real-time auto-coding so that practitioners can attest to the codes proposed by the algorithm for their dictated report.

## Data availability statement

Data supporting this study cannot be made immediately available due to legal agreements protecting the use of even anonymized patient reports, albeit that the protected health information (PHI) has been removed, as there remains a non-zero, remote possibility that even PHI-removed data can be used to identify cohorts of patients or subjects with rare conditions or combinations of disorders.

## Disclosure/potential conflicts of interest

3 of the 7 authors have ownership shares, leadership positions, and patents regarding the NLP algorithm implemented herein and the associated business entity (*Radnosis, VEEV Inc., Minneapolis, MN, USA*).

## CRediT authorship contribution statement

**Alexander M. McKinney:** Writing – review & editing, Writing – original draft, Software, Methodology, Investigation, Formal analysis, Data curation, Conceptualization. **Jessica A. Moore:** Writing – review & editing, Validation, Investigation, Formal analysis, Data curation. **Kevin Campbell:** Writing – review & editing, Software, Methodology, Investigation, Formal analysis, Data curation. **Thiago Braga:** Writing – review & editing, Validation, Investigation. **Jeffrey Rykken:** Writing – review & editing, Validation, Investigation, Formal analysis. **Bharathi Jagadeesan:** Writing – review & editing, Validation, Formal analysis. **Zeke J. McKinney:** Writing – review & editing, Writing – original draft, Validation, Supervision, Software, Project administration, Methodology, Investigation, Formal analysis, Data curation, Conceptualization.

## Declaration of competing interest

The authors declare the following financial interests/personal relationships which may be considered as potential competing interests:Alexander M. McKinney reports a relationship with VEEV, Inc. that includes: board membership and equity or stocks. Zeke J. McKinney reports a relationship with VEEV, Inc. that includes: board membership and equity or stocks. Kevin Campbell reports a relationship with VEEV, Inc. that includes: board membership and equity or stocks. Alexander McKinney has patent #US 17/173,123 issued to Assignee.
